# Detection of biological loads in sewage using the automated robot‐driven photoelectrochemical biosensing platform

**DOI:** 10.1002/EXP.20230128

**Published:** 2024-03-14

**Authors:** Yiming Zhang, Zhi Chen, Songrui Wei, Yujun Zhang, Hai Fu, Han Zhang, Defa Li, Zhongjian Xie

**Affiliations:** ^1^ Department of Laboratory Medicine Shenzhen Children's Hospital Shenzhen People's Republic of China; ^2^ Institute of Biomedical and Health Engineering Shenzhen Institute of Advanced Technology Chinese Academy of Sciences Shenzhen People's Republic of China; ^3^ International Collaborative Laboratory of 2D Materials for Optoelectronics Science and Technology of Ministry of Education Institute of Microscale Optoelectronics Shenzhen University Shenzhen People's Republic of China; ^4^ Shenzhen International Institute for Biomedical Research Shenzhen People's Republic of China

**Keywords:** CRISPR/Cas12a system, nucleic acid detection, photoelectrochemical biosensor, robot automation, sewage monitoring

## Abstract

Real‐time polymerase chain reaction (RT‐PCR) remains the most prevalent molecular detection technology for sewage analysis but is plagued with numerous disadvantages, such as time consumption, high manpower requirements, and susceptibility to false negatives. In this study, an automated robot‐driven photoelectrochemical (PEC) biosensing platform is constructed, that utilizes the CRISPR/Cas12a system to achieve fast, ultrasensitive, high specificity detection of biological loads in sewage. The Shennong‐1 robot integrates several functional modules, involving sewage sampling and pretreatment to streamline the sewage monitoring. A screen‐printed electrode is employed with a vertical graphene‐based working electrode and enhanced with surface‐deposited Au nanoparticles (NPs). CdTe/ZnS quantum dots (QDs) are further fabricated through the double‐stranded DNA (dsDNA) anchored on Au NPs. Using the cDNA template of Omicron BA.5 spike gene as a model, the PEC biosensor demonstrates excellent analytical performance, with a lower detection limit of 2.93 × 10^2^ zm and an outstanding selectivity at the level of single‐base mutation recognition. Furthermore, the rapid, accurate detection of BA.5 in sewage demonstrates the feasibility of the PEC platform for sewage monitoring. In conclusion, this platform allows early detection and tracking of infectious disease outbreaks, providing timely data support for public health institutions to take appropriate prevention and control measures.

## Graphical abstract

The current monitoring technologies for biological loads in sewage exhibit drawbacks, including low detection efficiency and insufficient accuracy. The authors' research team has developed an innovative approach that harnesses the power of the CRISPR/Cas12a system within an automated robot‐driven photoelectrochemical biosensing platform. This platform allows early detection and tracking of infectious disease outbreaks, providing timely data support for public health institutions to take appropriate prevention and control measures.

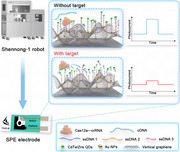

## 1 INTRODUCTION

Sewage, a unique interface between human beings and the natural environment, is generated by human activities and responsible for a considerable biological load, primarily from feces, saliva, and sputum. During the outbreak of the COVID‐19 pandemic, numerous studies reported the presence of a large quantity of COVID‐19 virus in sewage.^[^ ^1^, ^2^, ^3^ ^]^ In this context, biological load, particularly with respect to microbial pathogens, has attracted unprecedented attention.^[^ ^4^, ^5^ ^]^ On a global scale, microbial pathogens present a substantial obstacle to public health security and place a significant financial burden on the healthcare system, particularly given that three out of the top ten global health threats identified by the World Health Organization in 2019 were directly linked to pathogens: pandemic influenza, Ebola, and HIV. Sewage is an essential medium for capturing, accumulating, and transmitting pathogens;^[^ ^6^, ^7^, ^8^ ^]^ therefore, sewage has become an ideal medium for monitoring the pathogens of the entire community, and a powerful tool called wastewater‐based epidemiology has been developed to provide early warning for the emergence of disease and track the outbreak trend.^[^ ^5^, ^9^ ^]^ Various pathogens have been found in sewage worldwide, including nearly all types of bacteria that can cause human diseases, as well as viruses that have caused significant outbreaks in recent epidemics and pandemics.^[^ ^7^, ^10^ ^]^ Thus, it is extremely essential to establish a sewage monitoring platform for surveillance of various pathogens.

Real‐time polymerase chain reaction (RT‐PCR) is the most commonly employed molecular detection technique in sewage analysis, alongside other technologies utilized for this purpose.^[^ ^11^ ^]^ RT‐PCR, although widely regarded as the most sensitive technique for detecting nucleic acid due to its ability to rapidly replicate specific sequences, is susceptible to shortcomings such as false negative. In the context of SARS‐CoV‐2 detection, RT‐PCR assays have reported instances of inaccurate results, yielding false negatives even in confirmed infection cases.^[^ ^12^ ^]^ Also, the current RT‐PCR‐based detection methods require significant manpower and a prolonged testing period, which may not be able to meet the substantial testing capacity needed during widespread outbreaks.^[^ ^13^ ^]^ Furthermore, traditional sewage sampling, enrichment, concentration, nucleic acid extraction, and amplification lack the support of automation technology, requiring an investment of manpower and time, significantly reducing the timeliness of sewage monitoring. Therefore, developing a rapid, highly specific, and ultrasensitive surveillance platform for continuous monitoring of biological loads in sewage is necessary to obtain valuable information to effectively support disease treatment, prevention, and control.

Recently, clustered regularly interspaced short palindromic repeats (CRISPR)/CRISPR‐associated proteins (Cas) technology has led to its widespread recognition due to its potential application in molecular biological diagnosis.^[^ ^14^, ^15^ ^]^ Compared with traditional molecular diagnosis methods, the CRISPR/Cas nucleic acid detection system has higher sensitivity, specificity, and resolution.^[^ ^16^ ^]^ Moreover, the CRISPR/Cas nucleic acid detection system has a particular universality in biological nucleic acid detection.^[^ ^17^ ^]^ Different CRISPR/Cas systems, including DETECTR, HOLMES, and SHERLOCK, have been employed in the development of various nucleic acid detection methods.^[^ ^16^, ^18^, ^19^, ^20^, ^21^ ^]^ Recently, researchers have been incorporating CRISPR/Cas system with various detection platforms to enhance the sensitivity of nucleic acid detection methods. Kim et al. demonstrated the application of a CRISPR‐modified surface‐enhanced Raman scattering (SERS) assay for the identification of multi‐drug resistant bacteria, achieving a remarkable detection sensitivity as low as approximately 10 fm.^[^ ^22^ ^]^ In a similar vein, Hajian et al. developed a CRISPR/Cas9 system‐based graphene field‐effect transistor (FET) biosensing platform for the direct detection of unamplified target genes.^[^ ^23^ ^]^ This platform exhibited an impressive sensitivity of 1.7 fm and rapid response to the target sequence within a 15‐min timeframe. In our own studies, we developed CRISPR‐empowered surface plasmon resonance (SPR) and electrochemical biosensing platforms, presenting detection limits as low as 1.3 and 50 fm for unamplified DNA samples, respectively.^[^ ^24^, ^25^ ^]^

Photoelectrochemical (PEC) analysis has gained significant recognition as a versatile and promising analytical technology in various fields.^[^ ^26^, ^27^, ^28^ ^]^ Because the light emission source and electrical signal readout are completely separate, PEC sensors have notable benefits including high sensitivity, minimal background interference, rapid response time, and affordability.^[^ ^29^, ^30^ ^]^ The performance of PEC platform is significantly dependent on the intrinsic qualities of photoactive materials.^[^ ^31^ ^]^ Zinc sulfide (ZnS), characterized by good photocatalytic activity and serving as a promising photoactive matrix,^[^ ^32^, ^33^ ^]^ faces limitations in PEC sensor applications due to its wide band gap.^[^ ^34^ ^]^ The integration of other semiconductors as a photosensitization strategy can adjust the energy band structure through composite formation. This method can modify the electronic transport process and reduce the undesirable recombination of photogenerated electrons and holes, consequently enhancing the efficiency of photon‐to‐electron conversion.^[^ ^35^ ^]^ Cadmium telluride (CdTe), with narrow band gap and effective light‐harvesting properties, holds potential as a photosensitizer to enhance the photoactive properties of ZnS by facilitating the rapid separation of electrons and holes.^[^ ^36^, ^37^ ^]^

Therefore, an automated robot‐driven PEC biosensing platform based on a CRISPR/Cas12a system was constructed to meet the need for rapid, accurate detection of biological loads in sewage. The robot has the capacity to automate the complete process, which includes sampling, enrichment, concentration, nucleic acid extraction, and reverse transcription. The electrode used was a screen‐printed electrode, and the working electrode was based on vertical graphene with surface‐deposited Au nanoparticles (NPs). With the assistance of these Au NPs, double‐stranded DNA (dsDNA) was fixed on the electrode surface. Furthermore, CdTe/ZnS quantum dots (QDs) were fabricated on the electrode via an amide bond formed between the surface carboxylic acid groups of CdTe/ZnS QDs and amino groups modified at the 5′ end of dsDNA, and the resulting electrode denoted as CdTe/ZnS QDs–dsDNA/Au NPs/rGO (reduced graphene oxide) electrode was obtained. When the Cas12a–crRNA duplex was subjected to the target sequence, it was specifically recognized and combined with the target sequence, and trans‐cleavage activity of Cas12a was initiated. The dsDNA fixed on the electrode was nonspecifically cut, resulting in CdTe/ZnS QDs detaching from the electrode and reducing the photocurrent. The designed automated robot‐driven PEC biosensing platform showed excellent analytical performance in the ultrasensitive detection of Omicron BA.5 and potential application prospects in monitoring sewage.

## 2 RESULTS AND DISCUSSION

### 2.1 The underlying principle of an automated robot‐driven PEC biosensing platform

**FIGURE 1:**
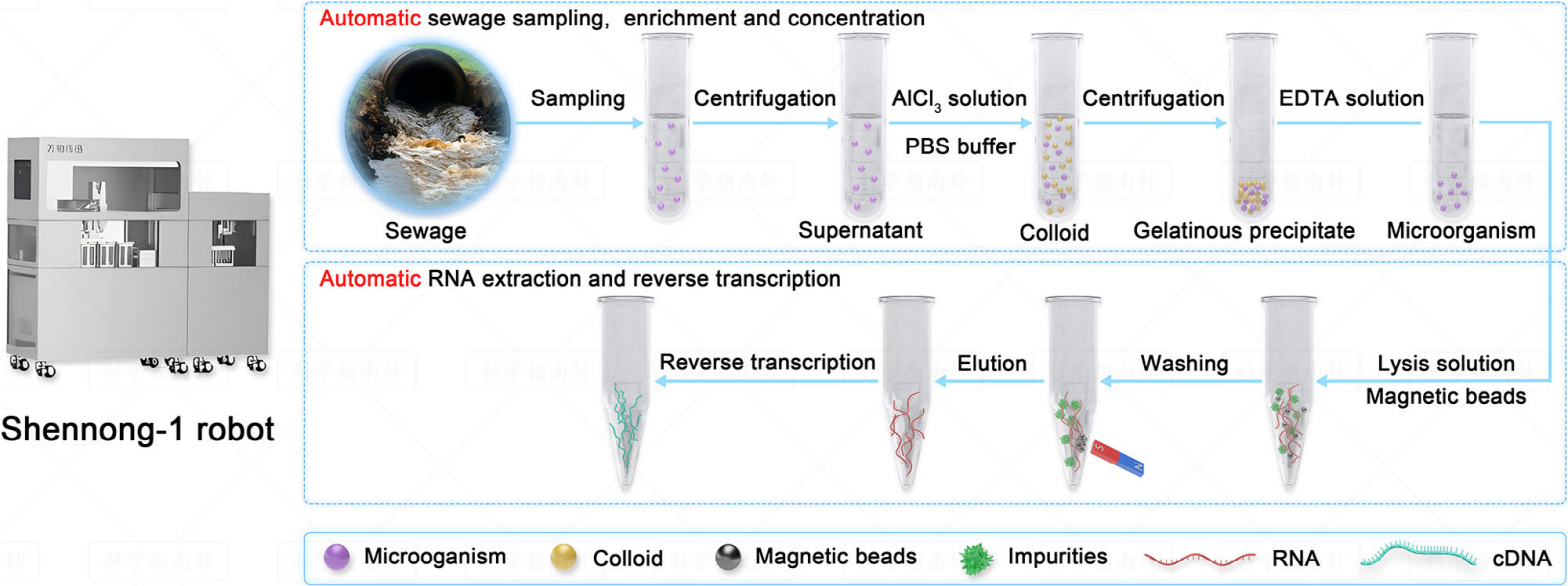
The Shennong‐1 robot for sewage sampling, enrichment, concentration, nucleic acid extraction, and reverse transcription.

**FIGURE 2:**
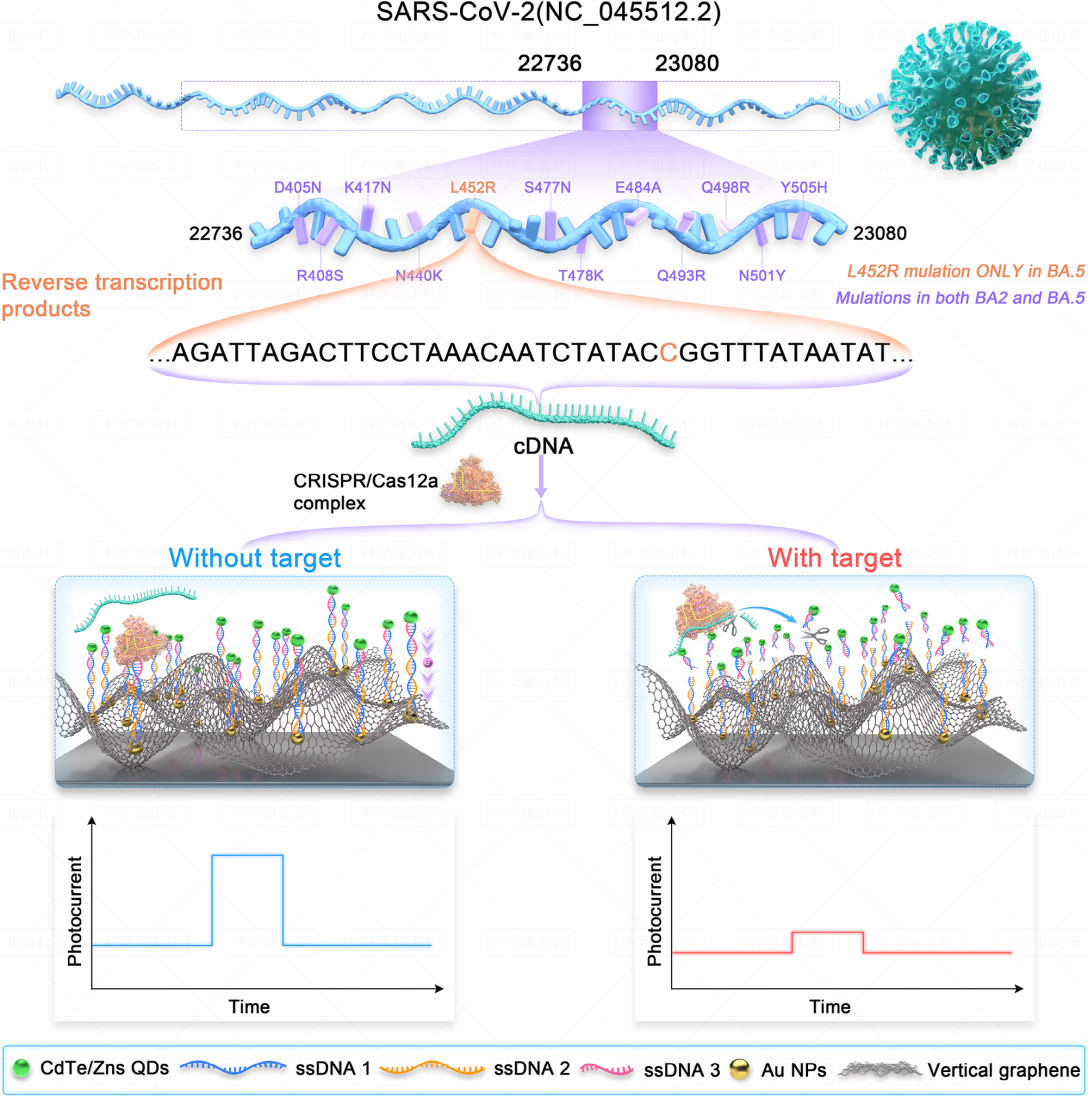
Principle of the PEC biosensor for the detection of cDNA using CRISPR/Cas12a system.

Considering Omicron BA.5 as an example, the principle of an automated robot‐driven PEC biosensing platform based on a CRISPR/Cas12a system is illustrated. The L452R mutation in the spike gene serves as an important marker for detecting Omicron BA.5 infection in the population. However, there is no protospacer adjacent motif (PAM) sequence near the L452R mutation, making it challenging to use Cas12a for detection of dsDNA amplification products associated with Omicron BA.5. A previous study reported that a Cas12a–crRNA duplex can specifically recognize and bind to single‐stranded DNA (ssDNA) in the absence of a PAM, initiating Cas12a's cleavage activity.^[^ ^16^ ^]^ Based on this report, we utilized the specificity of the Cas12a–crRNA duplex for recognizing target cDNA to detect the L452R mutation. To begin with, the automated Shennong‐1 robot was responsible for performing sewage sampling, enrichment, concentration, nucleic acid extraction, and reverse transcription (Figure 1). Subsequently, the resulting reverse transcription products were subjected to PEC analysis based on CRISPR/Cas12a‐mediated CdTe/ZnS QDs–dsDNA nonspecific cleavage (Figure 2). The surface of the Au NPs/rGO base electrode was modified with a CdTe/ZnS QDs–dsDNA reporter. The Cas12a–crRNA duplex was designed to precisely recognize Omicron BA.5 based on the complementarity between the target DNA and crRNA.^[^ ^38^ ^]^ In the absence of target cDNA, the cleavage activity of Cas12a remained inactive, causing CdTe/ZnS QDs‐dsDNA to remain intact on the modified electrode's surface and leading to a noticeable PEC signal. In the presence of target cDNA, the activity of Cas12a was initiated, resulting in trans‐cleavage activation. Consequently, non‐specific cutting of CdTe/ZnS QDs‐dsDNA occurred on the electrode surface, leading to a decline in the PEC signal. Therefore, the prepared CdTe/ZnS QDs‐assisted CRISPR–PEC biosensor successfully converts the target sequence recognition into the large‐scale cutting of dsDNA on the electrode followed by a change in photoelectric signal for ultrasensitive, highly specific PEC nucleic acid biosensing. In summary, the automated robot‐driven PEC biosensing platform constructed in this study has the potential for the rapid, ultrasensitive, and highly specific detection of Omicron BA.5 in sewage.

### 2.2 Morphological and compositional characterization of the CdTe/ZnS QDs–dsDNA/Au NPs/rGO electrode

**FIGURE 3:**
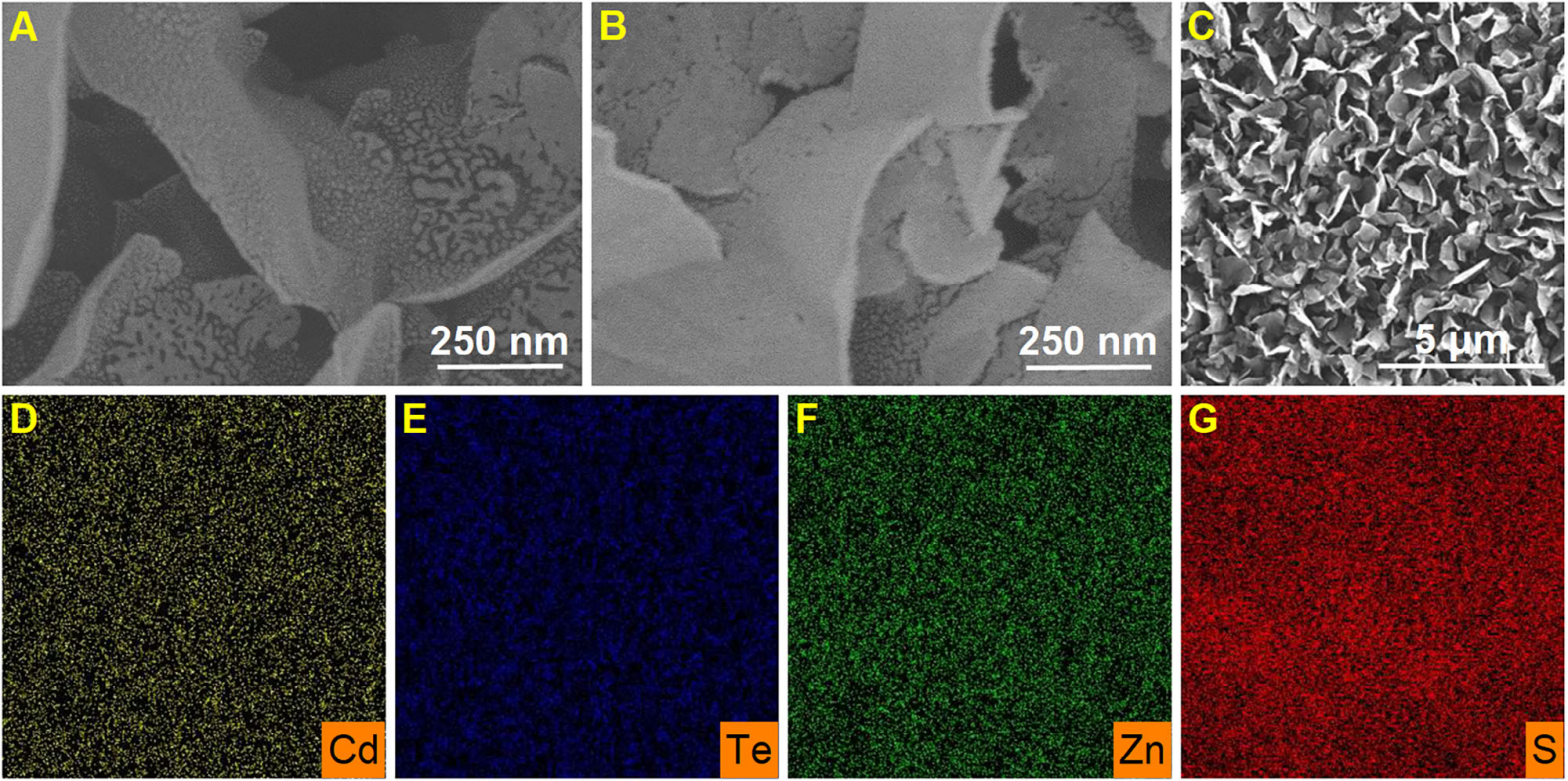
Morphological and compositional characterization of the electrodes. Scanning electron microscopy images of (A) the base electrode and (B) the assembled electrode. Elemental mappings of (C) the assembled electrode, (D) Cd, (E) Te, (F) Zn, and (G) S.

**FIGURE 4:**
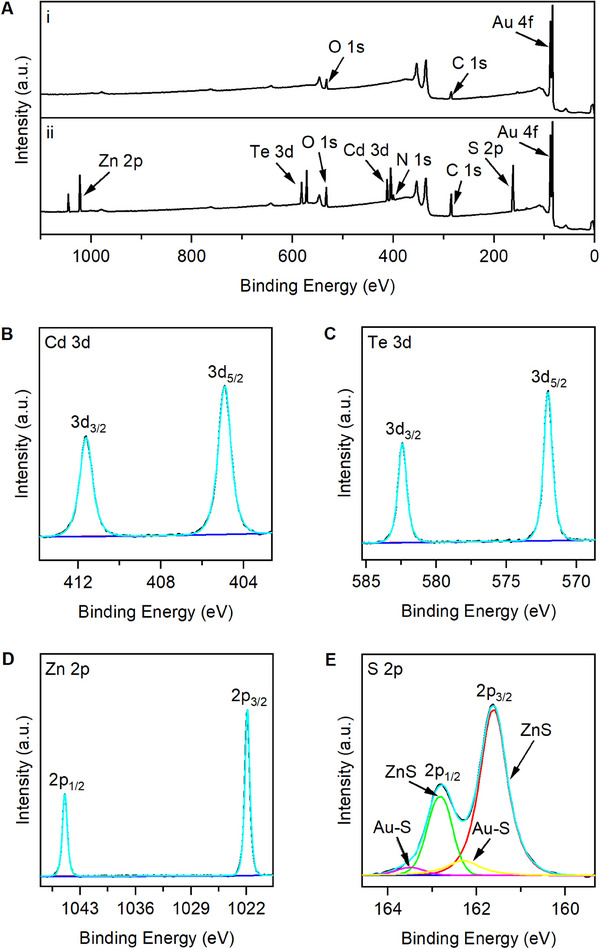
X‐ray photoelectron spectroscopy survey spectra of the electrodes. (A) X‐ray photoelectron spectroscopy survey spectra of (I) the base electrode and (II) the assembled electrode. High‐resolution XPS spectra of the assembled electrode for (B) Cd 3d, (C) Te 3d, (D) Zn 2p, and (E) S 2p.

The electrode morphology was assessed through scanning electron microscopy (SEM) measurements, both prior to and after the assembly process. Prior to assembly, the base electrode was composed of vertically oriented graphene with Au NPs deposited on its surface, which presented sharp edges and channels ranging in width from tens of nanometers to micrometers (Figure 3A). However, after assembly, the location of CdTe/ZnS QDs on the electrode surface was difficult to distinguish in SEM images due to their minimal particle size (Figure 3B). To address this issue, the elemental mapping was performed, confirming the homogeneous distribution of Cd, Te, Zn, and S on the CdTe/ZnS QDs–dsDNA/Au NPs/rGO electrode (Figure 3C–G). Furthermore, the surface element composition of the electrode was evaluated by X‐ray photoelectron spectroscopy (XPS). The assembled electrode exhibited increased C 1s and O 1s peak areas compared to the base electrode, which can be attributed to the presence of DNA probes and carboxyl groups on the surfaces of CdTe/ZnS QDs (Figure 4A). The N 1s peak observed in the assembled electrode was likely due to the presence of DNA, while the appearance of Cd 3d, Te 3d, Zn 2p, and S 2p peaks were attributed to the CdTe/ZnS QDs (Figure 4A). Furthermore, the characteristic peaks of CdTe were observed in the Cd 3d spectra (Figure 4B) at 411.6 eV and 404.9 eV, as well as in the Te 3d spectra (Figure 4C) at 582.4 eV and 572.0 eV. Characteristic peaks of ZnS were identified in the Zn 2p spectra (Figure 4D) at 1044.9 eV and 1022.0 eV, as well as in the S 2p spectra (Figure 4E) at 162.8 eV and 161.6 eV. It should be pointed out that the peaks at 163.5 eV and 162.3 eV were derived from Au─S bonds.^[^ ^39^ ^]^ All of the physical measurements and analysis described above confirmed the successful assembly of the CdTe/ZnS QDs–dsDNA/Au NPs/rGO electrode.

### 2.3 The design of the CRISPR/Cas12a system

**FIGURE 5:**
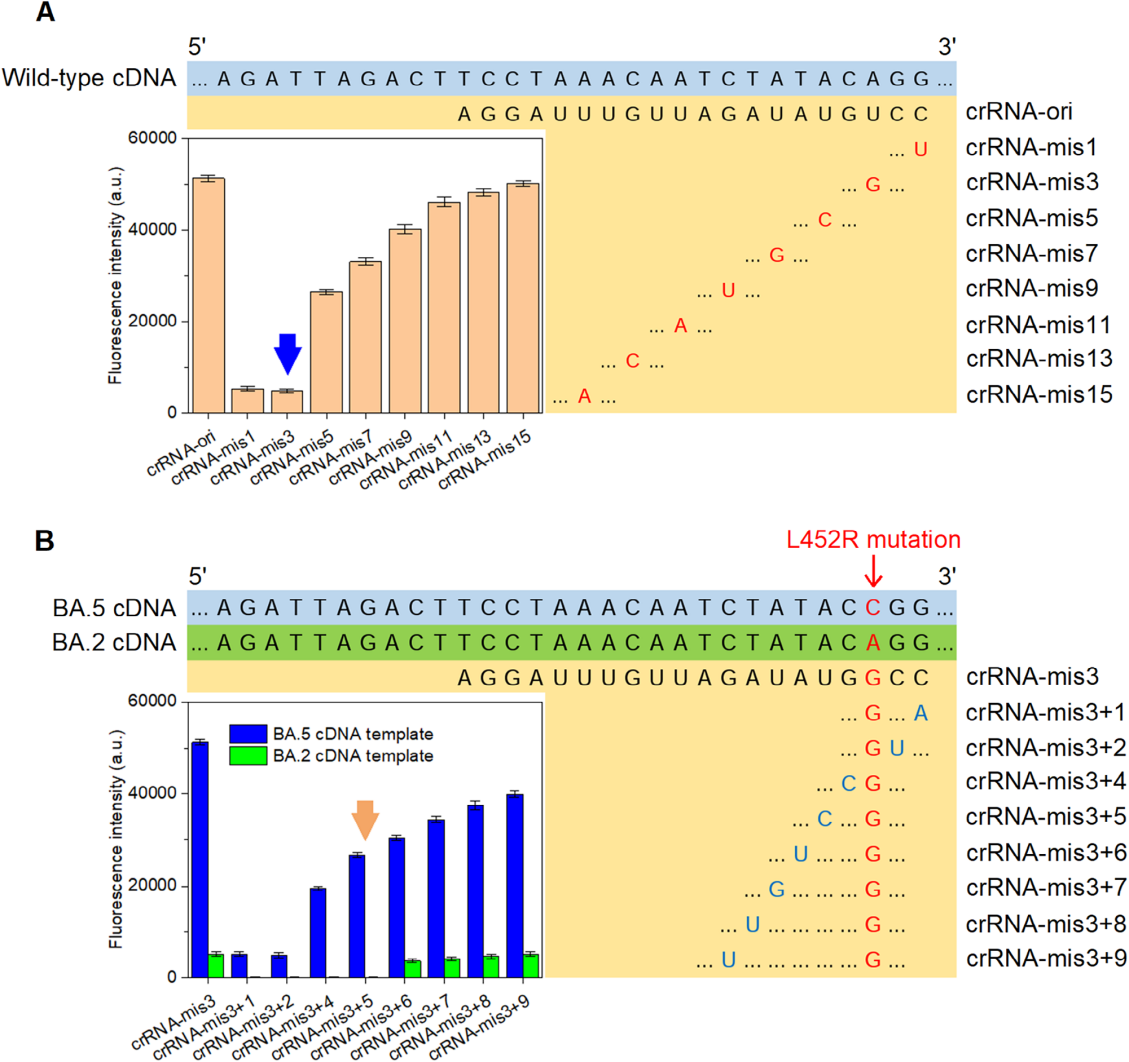
Optimization of crRNAs. Fluorescence analysis of crRNAs introduced with (A) a single mismatch at different sites targeting the SARS‐CoV‐2 wild‐type cDNA template and (B) a dual mismatch at different sites targeting the BA.2 and BA.5 cDNA templates.

The Cas12a protein functions as molecular “scissors”, precisely cleaving ssDNA sequences. These DNA‐targeting crRNAs bind with Cas12a, forming a complex known as Cas12a‐crRNA.^[^ ^40^, ^41^ ^]^ This complex acts as intelligent scissors, capable of cleaving the intended DNA target while sparing non‐targets, even when there's a single base mismatch. The precision of the CRISPR/Cas12a system heavily relies on meticulous design and selection of crRNA for specific applications.^[^ ^42^, ^43^ ^]^ To evaluate the system's specificity, we investigated the binding of crRNAs with mismatched bases at various positions to the original SARS‐CoV‐2 cDNA templates, with a concentration of 1 nm (as shown in Figure 5A). Notably, introducing a mismatch at either the 1st or 3rd base of the protospacer (crRNA‐mis1 and crRNA‐mis3) significantly diminished fluorescence signals, though not to a negligible level. As the mismatch was positioned further from the initiation site of the protospacer, the reduction in fluorescence became less pronounced. The impact of crRNA mismatches at positions 11 to 15 resembled that of the original crRNA. Remarkably, a mismatch at the 3rd site of the protospacer yielded the most minimal trans‐cleavage activity. Based on these findings, we propose that the SNP site, such as the L452R mutation, could be strategically positioned at the 3rd site of the protospacer within the crRNA sequence. This arrangement would maximize the differentiation between on‐target and off‐target samples. However, it's crucial to note that the off‐target trans‐cleavage activity does affect the accuracy of detecting the L452R mutation.

To address this challenge, a novel approach termed “dual mismatch” was implemented in the subsequent experiment (Figure 5B). For the BA.5 cDNA template, crRNA‐mis3 was identified as the appropriate crRNA sequence. The BA.2 cDNA template was considered analogous to the “wild‐type” sequence due to the absence of mutation sites within this range. An additional mismatch was introduced in the 1st to 9th sites of the protospacer, resulting in a series of crRNAs labeled as “crRNA‐mis3 + 1” to “crRNA‐mis3 + 9”. Each of these modified crRNAs was tested against both the BA.2 and BA.5 cDNA templates. When mismatches were introduced at positions 1 to 5 of the protospacer, almost no detectable fluorescence signal was observed during the assay with the BA.2 template. However, a mismatch at the 5th site still resulted in a relatively strong fluorescence signal when tested against the BA.5 template (approximately 52.27% of the signal from a perfect match). Based on these outcomes, the crRNA‐mis3 + 5 emerged as the optimal crRNA for targeting BA.5 cDNA sequences with the capability to discern single‐nucleotide changes. Following the successful design of the CRISPR/Cas12a system for enhanced cDNA sequence specificity, a robust PEC platform was established to further achieve high sensitivity in subsequent steps.

### 2.4 Electrochemical impedance spectroscopy and PEC characterization of the PEC biosensor

**FIGURE 6:**
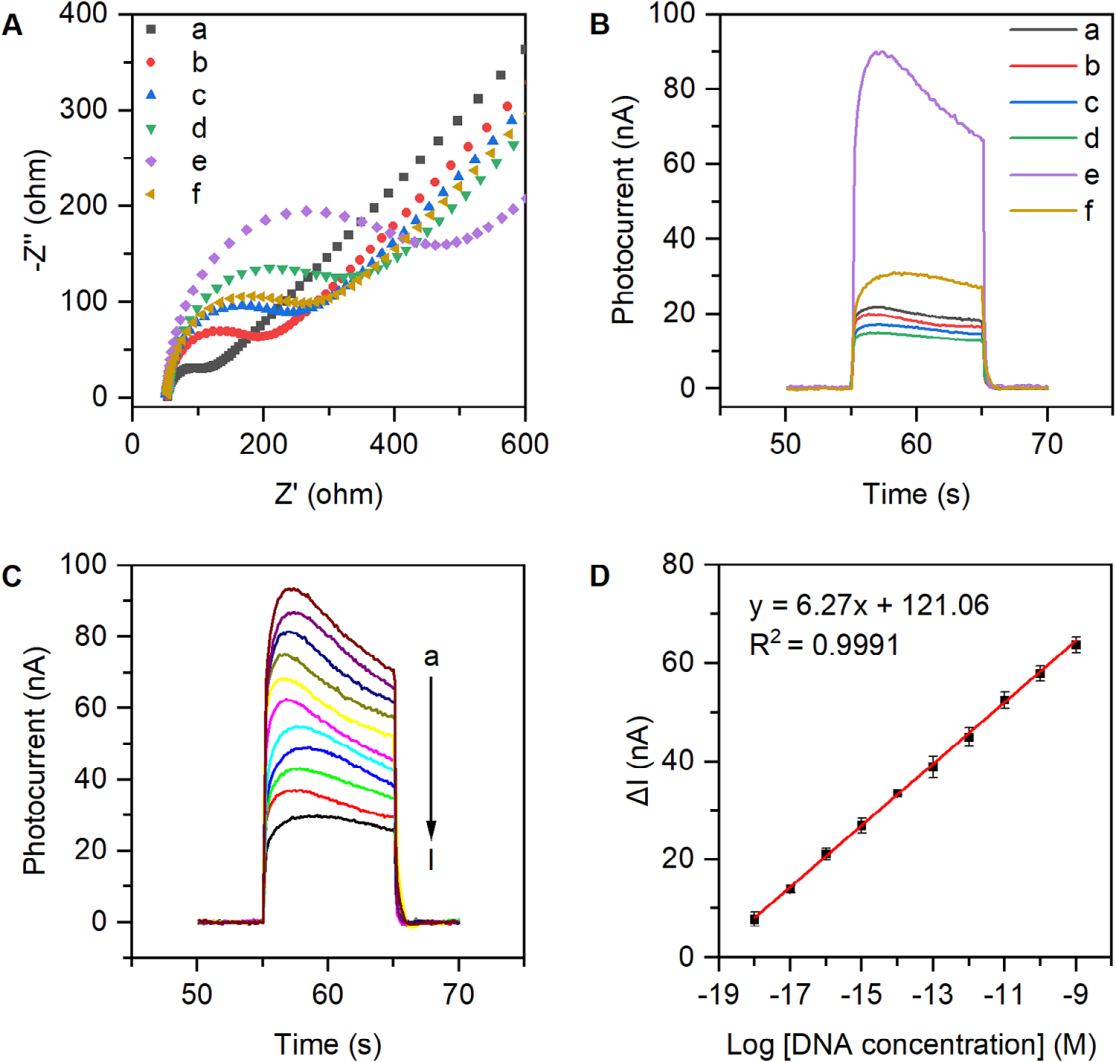
Electrochemical and PEC characterization, and detection performance of the fabricated biosensor. (A) Stepwise electrochemical impedance spectroscopy and (B) photocurrent response analysis of the fabricated PEC biosensor (a: Au NPs/rGO; b: ssDNA/Au NPs/rGO; c: MCH/ssDNA/Au NPs/rGO; d: MCH/dsDNA/Au NPs/rGO; e: CdTe/ZnS QDs/MCH/dsDNA/Au NPs/rGO; f: CdTe/ZnS QDs/MCH/dsDNA/Au NPs/rGO + Cas12a–crRNA–target DNA complex). (C) PEC responses of the biosensor to various concentrations of target DNA (a: 0; b: 1.0 am; c: 10.0 am; d: 100.0 am; e: 1.0 fm; f: 10.0 fm; g: 100.0 fm; h: 1.0 pm; i: 10.0 pm; j: 100.0 pm; and k: 1.0 nm). (D) Relationship between the change in signal (Δ*I*) and the logarithm of the concentration of target DNA. Error bars represent the standard deviation of triplicate measurements. Cas12a: CRISPR‐associated protein 12a; crRNA: CRISPR RNA; dsDNA: double‐stranded DNA; MCH: 6‐mercaptohexanol; NPs: nanoparticles; QDs: quantum dots; rGO: reduced graphite oxide; ssDNA: single‐stranded DNA.

Electrochemical impedance spectroscopy (EIS) is employed as a valuable technique to investigate the electrode interface, enabling the monitoring of the sequential assembly process of PEC biosensors. EIS tests were carried out in a 5 mm K_3_Fe(CN)_6_/K_4_Fe(CN)_6_ (1:1) solution with 0.1 m KCl serving as the supporting electrolyte. The tests were conducted at a bias potential of 0.14 V, employing a frequency range of 10^−2^−10^5^ Hz and an amplitude of 5 mV. Figure 6A showed that among these electrodes, the Au NPs/rGO electrode presented the most negligible interfacial electron transfer impedance value (*R_et_*) of about 84.95 Ω (curve a), which can be ascribed to the exceptional conductivity of vertical graphene and Au NPs. When the probe ssDNA1, 6‐mercaptohexanol (MCH), and ssDNA1 complementary chains (ssDNA2 and ssDNA3) were fabricated in turn on the electrode surface, the *R_et_* value gradually increased (curves b, c, and d, respectively). These results were attributed to the high steric hindrance and low conductivity of biological macromolecules (DNA, RNA) and MCH.^[^ ^44^ ^]^ The introduction of CdTe/ZnS QDs further hindered electron transfer (curve e) due to the steric hindrance effect of CdTe/ZnS QDs and their low conductivity. After incubating the electrode with the Cas12a–crRNA–target DNA complex, the *R_et_* value decreased again (curve f). This decline happened because the Cas12a–crRNA duplex indiscriminately cut the DNA probe on the electrode surface, which resulted in a reduction in both the quantities of CdTe/ZnS QDs and DNA, leading to a decrease in the steric hindrance effect and an increase in conductivity on the electrode surface.

The PEC response to electrode surface modifications was continuously monitored to assess the construction process of the proposed biosensor.^[^ ^45^, ^46^ ^]^ In Figure 6B, the Au NPs/rGO electrode demonstrated a noticeable photocurrent response (curve a) as a result of the SPR effect. In the case of SPR excitation, the energetic hot electrons generated in Au NPs can be directly transferred to vertical graphene and the external circuit, causing photocurrent generation.^[^ ^47^, ^48^ ^]^ When the probe ssDNA1, MCH, and ssDNA1 complementary chains (ssDNA2 and ssDNA3) were fabricated in turn on the electrode surface, the photocurrent signal gradually decreased through each of these steps (curves b, c, and d, respectively), owing to the high steric hindrance and low conductivity of DNA and MCH. After CdTe/ZnS QDs were fabricated on the MCH/dsDNA/Au NPs/rGO electrode, the photocurrent signal significantly increased (curve e) due to the excellent photoelectric characteristics of CdTe/ZnS QDs. When the electrode was incubated with the Cas12a–crRNA–target DNA complex, the photocurrent signal decreased again (curve f), because the Cas12a–crRNA duplex nonspecifically cut the DNA probe on the electrode surface, resulting in a decreased quantity of CdTe/ZnS QDs and subsequently a decrease in the photocurrent originating from CdTe/ZnS QDs. In summary, the successful fabrication of the biosensor was confirmed by all results obtained from EIS and PEC characterization.

### 2.5 Detection performance of PEC biosensor

Under the constructed experimental conditions, the potential of the PEC biosensor to detect Omicron BA.5 was explored by measuring the PEC response at various cDNA template concentrations. Figure 6C showed that over a concentration range from 0 to 1.0 nm, the photocurrent was monotonically smaller at higher concentrations of the Omicron BA.5 cDNA template. Furthermore, a strong correlation was found between the change in photocurrent (∆*I*, the difference between photocurrent levels with and without cDNA) and the logarithm of cDNA template concentration (Figure 6D). The figure illustrated the linear regression equation as Δ*I* = 6.27 log [DNA] + 121.06, which exhibited an *R* ^2^ value of 0.9991. The error bars in the figure represented the standard deviation σ calculated from three independent measurements. According to the 3*δ*/*K* method, the estimated detection limit was 2.93 × 10^2^ zm.^[^ ^49^, ^50^ ^]^ In this case, the standard deviation was computed based on six measurements of the background signal. In contrast to previous DNA detection strategies (Table S3), this PEC biosensor demonstrated a superior lower detection limit and a broader linear range.

### 2.6 Selectivity, stability, and reproducibility

For practical biosensors, it is vital to have outstanding selectivity, stability, and reproducibility. To investigate the selectivity of PEC biosensors, a range of potential interferents including Omicron BA.2, Wild‐type, MERS, H_1_N_1_, H_3_N_2_, Influenza B, and HRSV were utilized.

**FIGURE 7:**
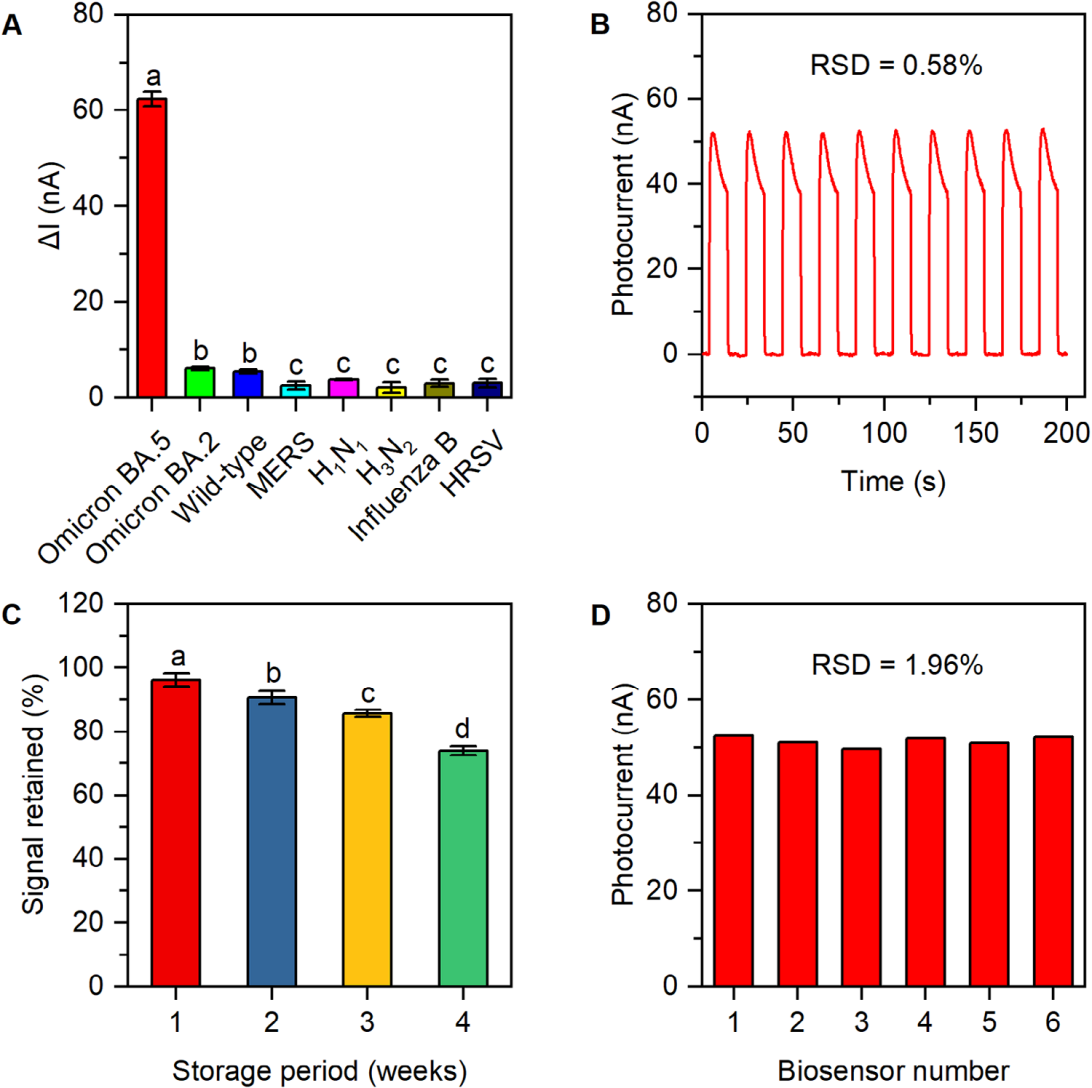
Selectivity, stability, and reproducibility of the biosensor. (A) Selectivity of the PEC biosensor incubated with various sequences: Omicron BA.5, Omicron BA.2, Wild‐type, MERS, H_1_N_1_, H_3_N_2_, Influenza B, and HRSV. (B) Photocurrent stability of the biosensor under consecutive off−on−off illumination for 10 cycles. (C) Responses of the PEC biosensor's photocurrent after different storage periods. (D) Reproducibility of the fabricated PEC biosensor. Columns with different superscript letters show significant differences (*p* < 0.05).

The selectivity of the PEC biosensor was determined by holding the cDNA concentration constant at 1.0 nm and comparing the ∆*I* value produced by the Omicron BA.5 cDNA template with the ∆*I* values produced by these potential interferents. According to Figure 7A, the photocurrent change generated by the Omicron BA.5 cDNA template was significantly greater compared to other potential interferents, demonstrating the notable selectivity of the PEC biosensor.

The investigation of the PEC biosensor's stability was also conducted. The photocurrent of the biosensor was evaluated in the detection buffer through multiple consecutive off–on–off irradiation cycles (10 cycles, 200 s; Figure 7B). The photocurrent response remained largely unchanged throughout the cycle test, with a minimal relative standard deviation (RSD) of only 0.58%, indicating the high stability and reliability of the PEC signal. To further study the biosensor's stability, a long‐term storage stability study was performed (Figure 7C). Upon storage in a dark and humid environment at 4°C for 1, 2, 3, and 4 weeks, the PEC biosensor exhibited photocurrent retention rates of 96.20%, 90.66%, 85.63%, and 73.99% of its initial response, highlighting its exceptional stability. The reproducibility of the PEC biosensor was evaluated by measuring the photocurrents of six biosensors assembled from the same batch and comparing their results. The photocurrents obtained from the six individual PEC biosensors exhibited negligible variation (RSD = 1.96%), demonstrating the remarkable reproducibility of the biosensor (Figure 7D).

### 2.7 Real sample analysis

**FIGURE 8:**
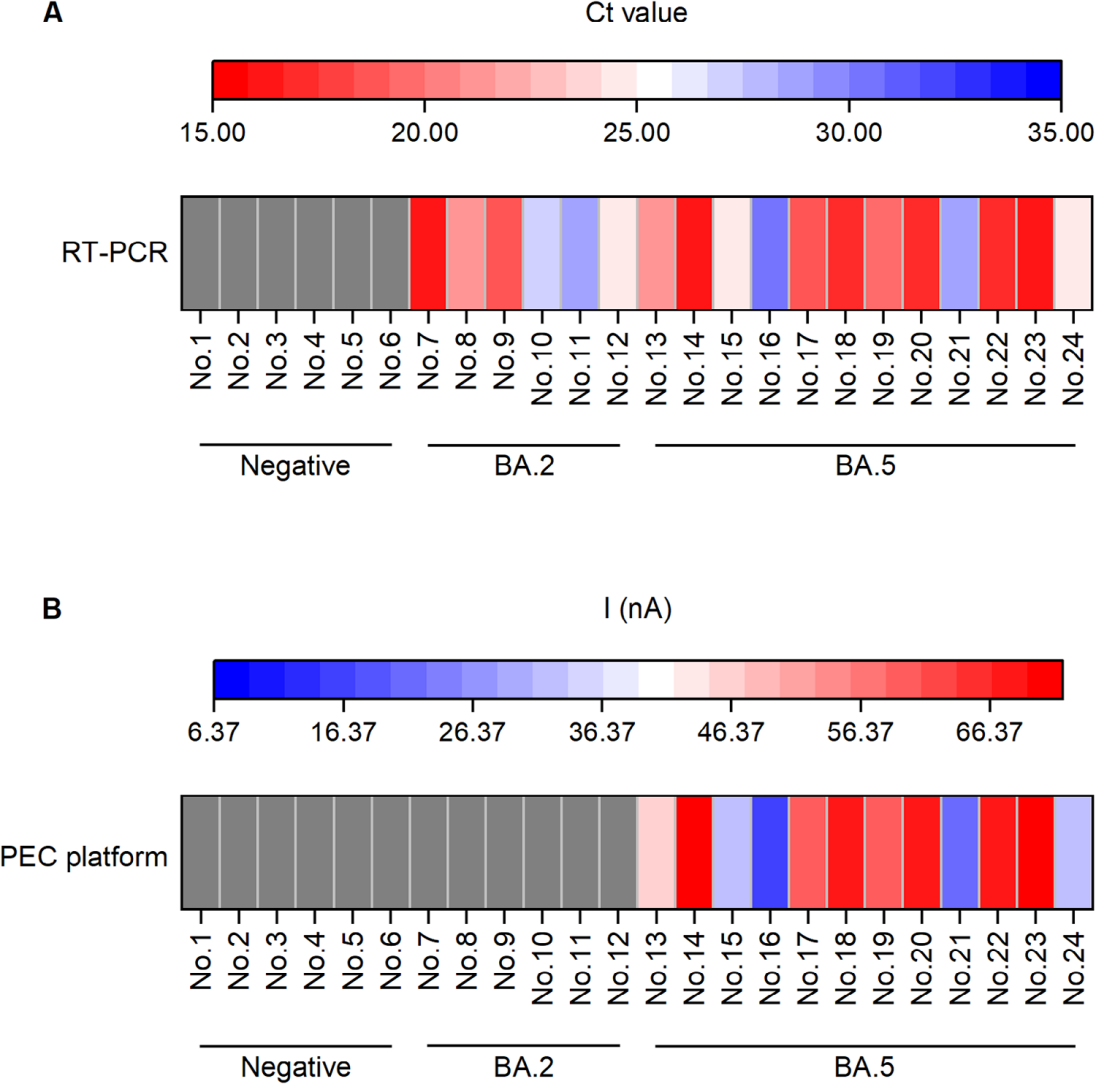
Evaluation of the PEC biosensing platform by real sample analysis. Detection of the L452R mutation in the spike gene of Omicron BA.5 in 24 sewage samples via (A) RT‐PCR and (B) the proposed biosensor.

To assess the sensitivity and specificity of the PEC sensing platform for detecting the BA.5 variant in sewage, a practical sample analysis was conducted. Initially, 24 sewage samples collected by automated robots were identified using RT‐PCR and Sanger sequencing. The sequencing results revealed that among the collected sewage samples, only the BA.2 and BA.5 variants were detected. Specifically, there were 6 negative samples, 6 samples positive for the BA.2 variant, and 12 samples positive for the BA.5 variant. The RT‐PCR analysis for the 18 positive sewage samples (Figure 8A) showed positive results, with Ct values ranging from 15 to 31. Conversely, the results for the 6 negative sewage samples were all negative. Additionally, using the same categorized samples, the PEC sensing platform was employed to detect the L452R mutation site (unique to the BA.5 variant and absent in the BA.2 variant). The positive threshold was defined as a Δ*I* value of 6.37 nA, representing the average of the blank signals plus triple its standard deviation. Positive results (Δ*I *> 6.37 nA) were observed exclusively in the samples containing the BA.5 variant (Figure 8B). These findings demonstrate that the PEC sensing platform can specifically monitor the crucial L452R variant of SARS‐CoV‐2. In summary, the PEC sensing platform demonstrates remarkable sensitivity and specificity in detecting the BA.5 variant in sewage.

### 2.8 PEC reaction mechanism

**FIGURE 9:**
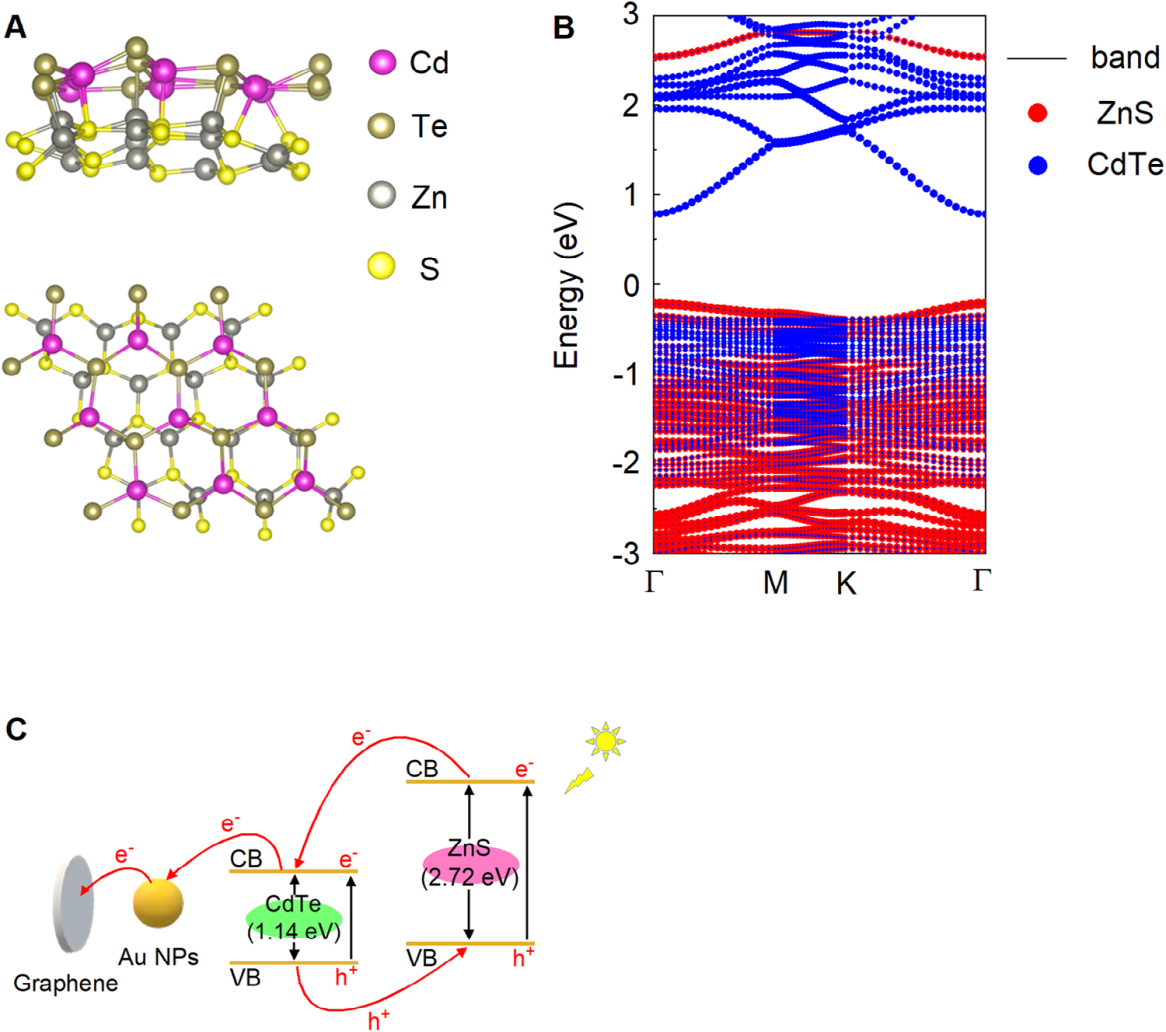
PEC reaction mechanism elucidated via density functional theory. (A) Top view and front view of the CdTe/ZnS QDs crystal structure model. (B) Calculated band structures of the CdTe/ZnS QDs. (C) The specific transfer mechanism of charge carriers.

In order to elucidate the PEC reaction mechanism of the constructed PEC sensing platform, a thorough investigation into the photogenerated carrier behavior of CdTe/ZnS QDs was conducted using first‐principle calculations based on density functional theory. The crystal and band structures of CdTe/ZnS QDs were depicted in Figures 9A and 9B, respectively. The conduction band (CB) and valence band (VB) energy levels of CdTe were found to be approximately 0.80 and −0.34 eV, respectively. Besides, for ZnS, the CB and VB energy levels were approximately 2.53 and −0.19 eV. The energy gaps of CdTe and ZnS were estimated to be 1.14 and 2.72 eV, respectively. Consequently, CdTe/ZnS QDs exhibit a type‐II heterostructure, resulting in the effective spatial separation of photoexcited electrons and holes. This spatial separation leads to prolonged carrier lifetimes due to reduced recombination rates. Based on these research findings, CdTe/ZnS QDs exhibit exceptional photoelectric response characteristics, which significantly enhance the sensitivity of the constructed biosensor.

The accompanying Figure 9C illustrates the specific mechanism of charge carrier transfer. Upon illumination, electrons from the VB of the semiconductors transition to the CB, resulting in an accumulation of holes in the VB. Subsequently, there is an electron transfer from ZnS to CdTe, while concurrently, holes are transferred from CdTe to ZnS due to the existing potential difference, thus realizing efficient transfer of photo‐generated carriers. Meanwhile, photoexcited electrons undergo a transfer process from the CB of CdTe to Au NPs. Ultimately, the electrons are conveyed to vertically aligned graphene, resulting in a remarkable photocurrent response.

## 3 CONCLUSIONS

In this study, a novel automated robot‐driven PEC biosensing platform, integrating a CRISPR/Cas12a system and PEC analysis, facilitated rapid, ultrasensitive, highly specific detection of biological loads in sewage. By harnessing the remarkable conductivity and extensive specific surface area offered by vertical graphene, the excellent photoelectric characteristics of CdTe/ZnS QDs, and the high specificity and efficient trans‐cleavage activity of the Cas12a–Cas system, the developed PEC biosensor exhibited outstanding analytical capabilities. The biosensor demonstrated an extensive linear range spanning from 1.0 am to 1.0 nm, a remarkable lower detection limit of 2.93×10^2^ zm, and exceptional selectivity at the level of single‐base mutation recognition. Furthermore, the Shennong‐1 robot was employed to automate sewage sampling, enrichment, concentration, nucleic acid extraction, and reverse transcription, expediting the analysis of Omicron BA.5 in sewage. This innovative robot‐driven PEC biosensing platform promises swift, ultrasensitive, and highly precise detection of Omicron BA.5, making it an excellent candidate for future applications of sewage monitoring for biological loads.

## 4 MATERIALS AND METHODS

### 4.1 Materials and reagents

K_3_Fe(CN)_6_, K_4_Fe(CN)_6_, KCl, 1‐ethyl‐3‐(3‐dimethylaminopropyl)carbodiimide (EDC), and *N*‐hydroxysuccinimide (NHS) were purchased from Aladdin Biotechnology Co., Ltd. (Shanghai, China). Carboxylated CdTe/ZnS QDs were purchased from Ruixi Biotechnology Co., Ltd. (Xi'an, China). Phosphate‐buffered saline (PBS) buffer (0.1 m, pH 7.4) and 2‐(*N*‐morpholino)ethanesulfonic acid (MES) buffer (0.1 m, pH 6.0) were purchased from Phygene Biotechnology Co., Ltd. (Fuzhou, China). Tris(2‐carboxyethyl)phosphine hydrochloride (TCEP), ethylenediaminetetraacetic acid (EDTA), and MCH were purchased from Macklin Biochemical Co., Ltd. (Shanghai, China). Cas12a, 10× Cas12a Reaction Buffer, and fluorescein amidite—single‐stranded DNA (FAM‐ssDNA) reporter were purchased from EZassay Biotechnology Co., Ltd. (Shenzhen, China). Nucleic acid extraction kit (Magnetic Beads Method) was purchased from Zhongyuan Biotechnology Co., Ltd. (Chongqing, China). PrimeScript RT reagent kit was purchased from TaKaRa Biotechnology Co., Ltd. (Dalian, China). HiScribe T7 High Yield RNA Synthesis Kit, Monarch RNA Cleanup Kit, and DNase I were purchased from New England Biolabs Inc. (Ipswich, MA, USA). The construction of plasmids carrying the designed dsDNA templates and the synthesis of crRNA and ssDNA (Table S1) were performed by Sangon Biotech Co., Ltd. (Shanghai, China).

### 4.2 Apparatus

The PEC test system (PEC1000, Beijing Perfectlight Technology Co., Ltd., China), equipped with an Xe lamp (PLS‐FX300HU, Beijing Perfectlight Technology Co., Ltd., China) and an electrochemical workstation (CHI 760E, Shanghai Chenhua Instrument Co., Ltd., China), was employed for PEC measurements. The base screen‐printed electrodes were purchased from Shenzhen Yick Xin Technology R&D Co., Ltd. (Shenzhen, China). The working electrode (2 mm diameter) was composed of vertical graphene with surface‐deposited Au NPs. The reference electrode employed was Ag/AgCl, and the counter electrode consisted of carbon paste.

SEM (SU‐70, Hitachi Co., Tokyo, Japan) was utilized to perform morphological characterization of the electrode. Energy dispersive spectroscopy (EDS; EX‐250, Horiba, Kyoto, Japan) was employed to characterize the distribution of chemical elements at the electrode interface. A surface analysis system (AXIS Ultra‐DLD, Kratos Analytical, Manchester, UK) equipped with monochromatic Al K*α* (1486.6 eV) radiation was utilized for conducting XPS analysis. The automated robot (Shennong‐1, Shenzhen Metasensing Technology Co., Ltd., China) was used for sewage sampling, enrichment, concentration, nucleic acid extraction, and reverse transcription.

### 4.3 Assembly of PEC biosensor

The base Au NPs/rGO electrode was first cleaned using sulfuric acid, potassium hydroxide, and nitric acid as described previously.^[^ ^51^ ^]^ The 5′‐thiol‐modified ssDNA1 was exposed to 10 µm TCEP for 10 min to avoid the generation of disulfide bonds and then diluted to 0.25 µm by addition of 10 mm Tris buffer containing 10 mm EDTA. Then, 200 µL of the 0.25 µm ssDNA1 solution was incubated onto the abovementioned electrode at room temperature for 1 h to obtain the ssDNA1/Au NPs/rGO electrode through Au─S bonding. Subsequently, the modified electrode was coated with 200 µL of 2 mm MCH for a duration of 30 min to block nonspecific adsorption sites and achieve a highly uniform surface. This step resulted in the formation of the MCH/ssDNA1/Au NPs/rGO electrode. Then 200 µL of PBS buffer containing ssDNA2 and 5′‐amino‐modified ssDNA3 with an equimolar concentration of 0.25 µm was added to the modified electrode and incubated at room temperature for 1 h to obtain an MCH/dsDNA/Au NPs/rGO electrode based on sequence complementarity. Subsequently, the CdTe/ZnS QDs suspension was stimulated by blending it with MES buffer (0.1 m, pH 6.0) consisting of 20 mm EDC and 10 mm NHS at a volume ratio of 1:9 for 1 h. Next, 200 µL of a suspension of activated CdTe/ZnS QDs was added to the modified electrode and incubated for 2 h to immobilize the CdTe/ZnS QDs via amide bonds. The obtained electrode was denoted as CdTe/ZnS QDs/MCH/dsDNA/Au NPs/rGO electrode, namely CdTe/ZnS QDs–dsDNA/Au NPs/rGO electrode. Following each step of the biosensor fabrication process, the electrode was subjected to three rinses with a washing buffer. Finally, the sensor was dried using nitrogen gas to prepare for PEC testing.

### 4.4 PEC detection

A Cas12a–crRNA duplex (100 nm Cas12a, 100 nm crRNA) was prepared by adding crRNA (20 µm) and Cas12a protein (20 µm) to the reaction buffer and incubating at room temperature for 10 min. Then 190 µL of this Cas12a–crRNA duplex was mixed with 10 µL of cDNA and incubated at room temperature for 10 min to facilitate the formation of the Cas12a–crRNA–target complex. 200 µL of the Cas12a–crRNA–target complex solution was added to an electrode surface covered with a CdTe/ZnS QDs–dsDNA reporter, followed by incubation at 37°C for 30 min. The electrode surface was then cleaned by immersing in PBS buffer for 5 min prior to PEC detection. PEC detection was carried out in PBS buffer (0.1 m, pH 7.4) at room temperature with the addition of 0.1 m KCl. The white light source was provided by an Xe lamp, which was cycled on and off at 10‐s intervals. The applied potential during the experiment was 0.3 V.

### 4.5 Sewage sampling and pretreatment via the Shennong‐1 robot

The Shennong‐1 robot consists of several functional modules, including automatic sewage sampling, automatic enrichment and concentration, automatic nucleic acid extraction, and automatic reverse transcription. In Shenzhen Third People's Hospital, the robots were installed in tributaries receiving sewage, mainly from areas where patients infected with Omicron BA.5 were isolated for treatment.

Enrichment and concentration of Omicron BA.5 in sewage was carried out following the aluminum hydroxide adsorption−precipitation method recommended by the standard WS/T 799–2022. Briefly, a 50 mL sewage sample was centrifuged at 2500 g for 30 min at 4°C. Then, 39.5 mL of supernatant, 0.5 mL of aluminum chloride solution (0.3 m), and 10 mL of PBS buffer (0.1 m, pH 6.4) were added to a 50 mL centrifuge tube and mixed evenly to form an aluminum hydroxide colloid. After 15 min of incubation at room temperature, centrifugation was performed at 1900 × *g* for 5 min at 4°C. The supernatant was discarded, and 0.20 g ± 0.01 g of disodium EDTA dihydrate was added to the remaining colloid. After treatment at 60°C for 6 min in a water bath, the resulting liquid with a volume of approximately 1 mL was obtained for RNA extraction.

The viral RNA extraction was performed using the nucleic acid extraction kit in accordance with the instructions provided by the manufacturer. Briefly, 15 µL of protease K, 500 µL of lysis solution, and 4 µL of magnetic bead solution were added to 200 µL of concentrated sewage, and the mixture was incubated at 65°C for 4 min. Subsequently, RNA was magnetically adsorbed for 1 min, after which the magnetic beads were washed with 600 µL detergent for 30 s. Finally, after RNA was washed off the magnetic beads by 100 µL eluent at 80°C over the course of 2 min, the magnetic beads were removed to obtain an RNA‐containing solution.

The reverse transcription of RNA extracted was conducted using the PrimeScript RT reagent kit, following the instructions provided by the manufacturer. To begin, a mixture comprising 10 µL of 5× gDNA (genomic DNA) Eraser Buffer, 5 µL of gDNA Eraser, and 35 µL of RNA template was prepared and subjected to an incubation at 42°C for 2 min. Then, the mixture was supplemented with 5 µL of PrimeScript RT Enzyme Mix I, 20 µL of RT Primer Mix, 20 µL of 5× PrimeScript Buffer 2, and 5 µL of RNase‐free distilled water (dH_2_O). After thorough mixing, the mixture was incubated at 37°C for 15 min, followed by a brief incubation at 85°C for 5 s. The cDNA products obtained were examined through PEC detection. The reagents and consumables required for each functional module were manually placed on the designed tray, after which the entire process was automatically completed by the robot.

### 4.6 Preparations of SARS‐CoV‐2 nucleic acid fragments

The specific crRNA used in the study was synthesized and listed in Table S1. The Cas12a crRNA consists of two functional parts: the scaffold region (UAAUUUCUACUAAGUGUAGAU) responsible for binding with the Cas12a protein, and a customized region, which is the reverse complement of the target sequence (≈20 bp). The customized region extends from the 3′ end of the scaffold to ensure specificity. The third base in the customized region was designated as the SNP site, and an additional mismatch base was introduced at the fifth position to maximize the difference in cleavage between on‐target and off‐target sequences. The L452R mutation (22917 T > G) is unique to the BA.5 Omicron variant and was used as a distinguishing feature between BA.5 and BA.2 variants. The target RNA sequence for detecting the L452R mutation on the spike (S) gene is provided in Table S2. The RNA templates from respiratory pathogens were synthesized through the transcription of the corresponding DNA templates carried on pUC57 plasmids using the HiScribe T7 High Yield RNA Synthesis Kit (Table S2). After the RNA synthesis, any remaining DNA templates were eliminated by treating the samples with DNase I. Finally, the resulting RNA templates were purified using the Monarch RNA Cleanup Kit and subsequently were reverse transcribed into cDNA via the PrimeScript RT reagent kit.

### 4.7 Analysis of mismatch in cDNA samples

To validate the specificity of each crRNA designed for targeting cDNA samples, a diagnostic CRISPR‐Cas12a assay was employed, utilizing fluorescence signals as indicators. The reaction mixture comprised 50 nm of Cas12a, 50 nm of crRNA, 1× Cas12a reaction buffer, 120 nm of ssDNA reporter, cDNA templates of wild‐type, BA.2, or BA.5 gene sequences, and pure water. Incubation was conducted at 37°C for 30 min, during which real‐time fluorescence measurements were performed with an excitation wavelength of 485 nm and emission wavelength of 528 nm.

### 4.8 Statistical analysis

The presented data are expressed as the mean ± standard deviation, with all measurements conducted across three parallel experiments (*n* = 3). Linear regression analysis was conducted to evaluate the goodness of fit (Pearson's correlation coefficient, *R* ^2^). Statistical analyses were executed through SPSS 20.0 software (SPSS Inc., Chicago, IL, USA). Variations among the different treatments were assessed employing a one‐way analysis of variance (ANOVA), followed by subsequent least significant difference (LSD) tests. Differences in mean values were deemed statistically significant when the calculated *p*‐value fell below 0.05.

## CONFLICT OF INTEREST STATEMENT

The authors declare no conflicts of interest.

## Supporting information

Supporting Information

## Data Availability

The data for this study are included in the article and the Supporting Information. Additional data supporting the findings of this study can be obtained from the corresponding authors upon request.
