# Supplementary material for: Detection of biological loads in sewage using the automated robot‐driven photoelectrochemical biosensing platform
Source: Exploration (Beijing). 2024 Mar 14;4(5):20230128. doi: 10.1002/EXP.20230128 (PMC11491307; doi:10.1002/EXP.20230128)
Supplement: Supplementary file 1 — Supporting Information [file EXP2-4-20230128-s001.pdf]

Supporting Information

Detection of biological loads in sewage using the automated robot-driven photoelectrochemical biosensing platform

Yiming Zhang,[1,2] *Zhi Chen,*[3] *Songrui Wei,*[3] *Yujun Zhang,*[1,2] *Hai Fu,*[1,2] *Han Zhang,*[3*] *Defa Li,*[1*] *Zhongjian Xie*[1,4*]

[1]Department of Laboratory Medicine, Shenzhen Children's Hospital, Shenzhen 518038, P.R. China

[2]Institute of Biomedical and Health Engineering, Shenzhen Institute of Advanced Technology, Chinese Academy of Sciences, Shenzhen 518055, P.R. China

[3]International Collaborative Laboratory of 2D Materials for Optoelectronics Science and Technology of Ministry of Education, Institute of Microscale Optoelectronics, Shenzhen University, Shenzhen 518060, P.R. China

[4]Shenzhen International Institute for Biomedical Research, Shenzhen 518116, P.R. China

*Corresponding author: hzhang@szu.edu.cn; dfl_szetyy@126.com; zjxie2011@163.com

Yiming Zhang, Zhi Chen and Songrui Wei contributed equally to this work.

Table S1. CRISRP-Cas12a-crRNA, ssDNA and ssDNA reporter used in this study. crRNA was designed according to the "CRISPR-SHERLOCK" method. Lower-case letters indicate the "scaffold" sequence for LbCas12a protein. Base labelled in RED indicate the single nucleotide polymorphism (SNP) site; base labelled in GREEN indicate the "deliberately" introduced mismatch.

Name	Sequence (5'-3')
crRNA-ori	uaauuucuacuaaguguagauCCUGUAUAGAUUGUUUAGGA
crRNA-mis1	uaauuucuacuaaguguagauUCUGUAUAGAUUGUUUAGGA
crRNA-mis3	uaauuucuacuaaguguagauCCGGUAUAGAUUGUUUAGGA
crRNA-mis5	uaauuucuacuaaguguagauCCUGCAUAGAUUGUUUAGGA
crRNA-mis7	uaauuucuacuaaguguagauCCUGUAGAGAUUGUUUAGGA
crRNA-mis9	uaauuucuacuaaguguagauCCUGUAUAUAUUGUUUAGGA
crRNA-mis11	uaauuucuacuaaguguagauCCUGUAUAGAAUGUUUAGGA
crRNA-mis13	uaauuucuacuaaguguagauCCUGUAUAGAUUCUUUAGGA
crRNA-mis15	uaauuucuacuaaguguagauCCUGUAUAGAUUGUAUAGGA
crRNA-mis3+1	uaauuucuacuaaguguagauACGGUAUAGAUUGUUUAGGA
crRNA-mis3+2	uaauuucuacuaaguguagauCUGGUAUAGAUUGUUUAGGA
crRNA-mis3+4	uaauuucuacuaaguguagauCCGCUAUAGAUUGUUUAGGA
crRNA-mis3+5	uaauuucuacuaaguguagauCCGGCAUAGAUUGUUUAGGA
crRNA-mis3+6	uaauuucuacuaaguguagauCCGGUUUAGAUUGUUUAGGA
crRNA-mis3+7	uaauuucuacuaaguguagauCCGGUAGAGAUUGUUUAGGA
crRNA-mis3+8	uaauuucuacuaaguguagauCCGGUAUUGAUUGUUUAGGA
crRNA-mis3+9	uaauuucuacuaaguguagauCCGGUAUAUAUUGUUUAGGA
ssDNA1	SH-CTTTACTCAACttattattACGAACATCAGG
ssDNA2	ataaGTTGAGTAAAG
ssDNA3	NH2-CCTGATGTTCGTaata
FAM-ssDNA reporter	FAM- CTCAACttattattACGAAC-BHQ

Table S2. Sequences from SARS-CoV-2 wild-type, Omicron BA.5, Omicron BA.2, Middle East respiratory syndrome-related coronavirus (MERS), Influenza A virus (H_1N_1/H_3N_2), Influenza B virus, and Human Respiratory Syncytial Virus (HRSV). The specific mutation in BA.5 variant (L452R: 22917 T > G) was labelled in RED. The mutation sites (D405N, R408S, K417N, N440K, S477N, T478K, E484A, Q493R, Q498R, N501Y, and Y505H, respectively), both in BA.2 and BA.5

variant, were labelled in GREEN.

Name	Inserted Sequences (5’-3’)
SARS-CoV-2 wild-type (NC_045512. 2, S gene, 22736-23080)	TTTACTAATGTCTATGCAGATTCATTTGTAATTAGAGGTGATGAAGTC<br>AGACAAATCGCTCCAGGGCAAACTGGAAAGATTGCTGATTATAATTA<br>TAAATTACCAGATGATTTTACAGGCTGCGTTATAGCTTGGAATTCTAA<br>CAATCTTGATTCTAAGGTTGGTGGTAATTATAATTACCTGTATAGATTG<br>TTTAGGAAGTCTAATCTCAAACCTTTTGAGAGAGATATTTCAACTGA<br>AATCTATCAGGCCGGTAGCACACCTTGTAATGGTGTTGAAGGTTTTA<br>ATTGTTACTTTCCTTTACAATCATATGGTTTCCAACCCACTAATGGTGT<br>TGGTTACCAA
SARS-CoV-2 BA.5	TTTACTAATGTCTATGCAGATTCATTTGTAATTAGAGGTAATGAAGTC<br>AGACAAATCGCTCCAGGGCAAACTGGAAATATTGCTGATTATAATTAT<br>AAATTACCAGATGATTTTACAGGCTGCGTTATAGCTTGGAATTCTAAC<br>AAGCTTGATTCTAAGGTTGGTGGTAATTATAATTACCGGTATAGATTG<br>TTTAGGAAGTCTAATCTCAAACCTTTTGAGAGAGATATTTCAACTGA<br>AATCTATCAGGCCGGTAACAAACCTTGTAATGGTGTTGCAGGGTTTA<br>ATTGTTACTTTCCTTTACGATCATATGGTTTCCGACCCACTTATGGTGT<br>TGGTCACCAA
SARS-CoV-2 BA.2	TTTACTAATGTCTATGCAGATTCATTTGTAATTAGAGGTAATGAAGTC<br>AGACAAATCGCTCCAGGGCAAACTGGAAATATTGCTGATTATAATTAT<br>AAATTACCAGATGATTTTACAGGCTGCGTTATAGCTTGGAATTCTAAC<br>AAGCTTGATTCTAAGGTTGGTGGTAATTATAATTACCTGTATAGATTGT<br>TTAGGAAGTCTAATCTCAAACCTTTTGAGAGAGATATTTCAACTGAA<br>ATCTATCAGGCCGGTAACAAACCTTGTAATGGTGTTGCAGGGTTTAA<br>TTGTTACTTTCCTTTACGATCATATGGTTTCCGACCCACTTATGGTGTT<br>GGTCACCAA
MERS (NC_019843. 3, 24275-24938)	TGTTAATATGGAAGCCGCGTATACTTCATCTTTGCTTGGCAGCATAGC<br>AGGTGTTGGCTGGACTGCTGGCTTATCCTCCTTTGCTGCTATTCCATT<br>TGCACAGAGTATCTTTTATAGGTTAAACGGTGTTGGCATTACTCAACA<br>GGTTCTTTCAGAGAACCAAAAGCTTATTGCCAATAAGTTTAATCAGG<br>CTCTGGGAGCTATGCAAACAGGCTTCACTACAACTAATGAAGCTTTT<br>CAGAAGGTTCAGGATGCTGTGAACAACAATGCACAGGCTCTATCCA<br>AATTAGCTAGCGAGCTATCTAATACTTTTGGTGCTATTTCCGCCTCTAT<br>TGGAGACATCATACAACGTCTTGATGTTCTCGAACAGGACGCCCAAA<br>TAGACAGACTTATTAATGGCCGTTTGACAACACTAAATGCTTTTGTTG<br>CACAGCAGCTTGTTCGTTCCGAATCAGCTGCTCTTTCCGCTCAATTG<br>GCTAAAGATAAAGTCAATGAGTGTGTCAAGGCACAATCCAAGCGTT<br>CTGGATTTTGCGGTCAAGGCACACATATAGTGTCCTTTGTTGTAAATG

	CCCCTAATGGCCTTTACTTCATGCATGTTGGTTATTACCCTAGCAACC<br>ACATTGAGGTTGTTTCTGCTTATGGTCTTTGCGATGCAGCTAACCCT
Influenza A virus, H_1N_1 (NC_026431.1)	ATGAGTCTTCTAACCGAGGTCGAAACGTACGTTCTTTCTATCATCCCG<br>TCAGGCCCCCTCAAAGCCGAGATCGCGCAGAGACTGGAAAGTGTCT<br>TTGCAGGAAAGAACACAGATCTTGAGGCTCTCATGGAATGGCTAAA<br>GACAAGACCAATCTTGTCACCTCTGACTAAGGGAATTTTAGGATTTG<br>TGTTCACGCTCACCGTGCCCAGTGAGCGAGGACTGCAGCGTAGACG<br>CTTTGTCCAAAATGCCCTAAATGGGAATGGGGACCCGAACAACATG<br>GATAGAGCAGTTAAACTATACAAGAAGCTCAAAAGAGAAATAACGT<br>TCCATGGGGCCAAGGAGGTGTCACTAAGCTATTCAACTGGTGCACTT<br>GCCAGTTGCATGGGCCTCATATACAACAGGATGGGAACAGTGACCA<br>CAGAAGCTGCTTTTGGTCTAGTGTGTGCCACTTGTGAACAGATTGCT<br>GATTCACAGCATCGGTCTCACAGACAGATGGCTACTACCACCAATCC<br>ACTAATCAGGCATGAAAACAGAATGGTGCTGGCTAGCACTACGGCA<br>AAGGCTATGGAACAGATGGCTGGATCGAGTGAACAGGCAGCGGAGG<br>CCATGGAGGTTGCTAATCAGACTAGGCAGATGGTACATGCAATGAGA<br>ACTATTGGGACTCATCCTAGCTCCAGTGCTGGTCTGAAAGATGACCT<br>TCTTGAAAATTTGCAGGCCTACCAGAAGCGAATGGGAGTGCAGATG<br>CAGCGATTCAAGTGATCCTCTCGTCATTGCAGCAAATATCATTGGGAT<br>CTTGCACCTGATATTGTGGATTACTGATCGTCTTTTTTTCAAATGTATT<br>TATCGTCGCTTTAAATACGGTTTGAAAAGAGGGCCTTCTACGGAAGG<br>AGTGCCTGAGTCCATGAGGGAAGAATATCAACAGGAACAGCAGAGT<br>GCTGTGGATGTTGACGATGGTCATTTTGTCAACATAGAGCTAGAGTA<br>A
Influenza A virus, H_3N_2 (U51247.1)	ATGAATCCAAATCAAAAGATAATAACAATTGGCTCTGTTTCTCTCACT<br>ATTGCCACAATATGCTGCCTTATGCAAATTGCCATCCTGGTAACTACT<br>GTAACATTACATTTCAAGCAATATGAATGCAACTCCCCCCCAAACAA<br>CCAAGTAATGCTGTGTGAACCAACAATAATAGAAAGAAACATAACA<br>GAGATAGTGTATCTGACCAACACCACCATAGAGAAAGAAGTATGCCC<br>CAAACTAGCAGAATACAGAAATTGGTCAAAGCCGCAATGTAAAATTA<br>CAGGATTTGCACCTTTTTCTAAGGACAATTCAATTCGGCTTTCCGCTG<br>GTGGAGACATTTGGGTGACAAGAGAACCTTATGTGTCATGCGATCCT<br>GGCAAGTGTTATCAATTTGCCCTTGGACAGGGAACAACACTAAACA<br>ACAGGCATTCAAATGACACAGTACATGATAGGACCCCTTATCGAACC<br>CTATTGATGAATGAGTTGGGTGTTCCATTTCATTTGGGAACCAAGCA<br>AGTGTGCATAGCATGGTCCAGCTCAAGTTGTCACGATGGAAAAGCAT<br>GGCTGCATGTTTGTGTAACTGGGCATGATGAAAATGCAACTGCTAGC<br>TTCATTTACGATGGGAGGCTTGTAGATAGTATTGGTTCATGGTCCAAA<br>AATATCCTCAGGACCCAGGAGTCGGAATGCGTTTGTATCAATGGAAC<br>TTGTACAGTAGTAATGACTGATGGAAGTGCTTCAGGAAGAGCTGATA

	CTAAAATACTATTCATTGAAGAGGGGAAAATCGCTCATATTAGCCCAT<br>TGTCAGGAAGTGCTCAGCATGTCGAGGAGTGCTCCTGTTATCCTCGA<br>TATCCTGGTGTCAGATGTGTCTGCAGAGACAACTGGAAAGGCTCCA<br>ATAGGCCCATCGTAGATATAAATGTGAAAGATTATAGCATTGTTTCCA<br>GTTATGTGTGCTCAGGACTTGTTGGAGACACAGCCAGAAAAAACGA<br>CAGCTCCAGCAGTAGCTATTGCCGGAATCCTAACAATGAGAAAGGG<br>AGTCATGGAGTGAAAGGCTGGGCCTTTGATGATGGAAATGATGTGTG<br>GATGGGAAGAACGATCAGCGAGAAGTTACGCTCAGGTGATGAAACC<br>TTCAAAGTCATTGGAGGCTGGTCCAAACCTAACTCCAAATTGCAGAT<br>AAATAGGCAAGTCATAGTTGACAGAGGTAATAGGTCCGGTTATTCTG<br>GTATTTTCTCTGTTGAAGGCAAAAGCTGCATCAATCGGTGCTTTTATG<br>TGGAGTTGATAAGGGGAAGGAAACAGGAAACTGAAGTCTGGTGGA<br>CCTCAAACAGTATTGTTGTGTTTTGTGGCACCTCAGGTACATATGGA<br>ACAGGCTCATGGCCTGATGGGGCGGACATCAATCTCATGCCTATATAA<br>A
Influenza B virus segement 7 (NC_002210.1)	AGCAGAAGCACGCACTTTCTTAAAATGTCGCTGTTTGGAGACACAAT<br>TGCCTACCTGCTTTCACTAATAGAAGATGGAGAAGGCAAAGCAGAA<br>CTAGCTGAAAAATTACACTGTTGGTTCGGTGGGAAAGAATTTGACCT<br>AGATTCTGCTTTGGAATGGATAAAAAACAAAAGGTGCCTAACTGATA<br>TACAAAAAGCACTAATTGGTGCCTCTATATGCTTTTTAAAACCCAAA<br>GACCAAGAAAGAAAAAGGAGATTCATCACAGAGCCCCTGTCAGGA<br>ATGGGAACAACAGCAACAAAGAAGAAAGGCCTAATTCTAGCTGAGA<br>GAAAAATGAGAAGATGTGTAAGCTTTCATGAAGCATTTGAAATAGCA<br>GAAGGCCACGAAAGCTCAGCATTACTATATTGTCTTATGGTCATGTAC<br>CTAAACCCTGAAAACTATTCAATGCAAGTAAAACTAGGAACGCTCTG<br>TGCTTTATGCGAGAAACAAGCATCGCACTCGCATAGAGCCCATAGCA<br>GAGCAGCAAGGTCTTCGGTACCTGGAGTAAGACGAGAAATGCAGAT<br>GGTTTCAGCTATGAACACAGCAAAGACAATGAATGGAATGGGAAAG<br>GGAGAAGACGTCCAAAAACTAGCAGAAGAGCTGCAAAACAACATT<br>GGAGTGTTGAGATCTCTAGGAGCAAGTCAAAAGAATGGAGAAGGA<br>ATTGCCAAAGATGTAATGGAAGTGCTAAAACAGAGCTCTATGGGAA<br>ATTCAGCTCTTGTGAGGAAATACTTATAATGCTCGAACCACTTCAGAT<br>TCTTTCAATTTGTTCTTTCATTTTATCAGCTCTCCATTTCATGGCTTGG<br>ACAATAGGGCATTTGAATCAAATAAAAAGAGGGGTAAACTTGAAAA<br>TACAAATAAGGAATCCAAATAAGGAGGCAATAAACAGAGAGGTGTC<br>AATTCTGAGACACAATTACCAAAAGGAAATCCAAGCCAAAGAAACA<br>ATGAAGAAAATACTCTCTGACAACATGGAAGTATTGGGTGACCACAT<br>AGTAGTTGAAGGGCTTTCAACTGATGAGATAATAAAAATGGGTGAAA<br>CAGTTTTGGAGGTGGAAGAATTGCAATGAGCCCAATTTTCACTGTAT<br>TTCTTACTATGCATTTAAGCAAATTGTAATCAATGTCAGTGAATAAAA<br>CTGGAAAAAGTGCGTTGTTTCTACT

HRSV, Human orthopneumo virus Subgroup A, (NC_038235.1)	GGGGCAAATACAAAGATGGCTCTTAGCAAAGTCAAGTTGAATGATAC<br>ACTCAACAAAGATCAACTTCTGTCATCCAGCAAATACACCATCCAAC<br>GGAGCACAGGAGATAGTATTGATACTCCTAATTATGATGTGCAGAAA<br>CACATCAATAAGTTATGTGGCATGTTATTAATCACAGAAGATGCTAAT<br>CATAAATTCACTGGGTTAATAGGTATGTTATATGCGATGTCTAGGTTAG<br>GAAGAGAAGACACCATAAAAATACTCAGAGATGCGGGATATCATGTA<br>AAAGCAAATGGAGTAGATGTAACAACACATCGTCAAGACATTAATGG<br>AAAAGAAATGAAATTTGAAGTGTTAACATTGGCAAGCTTAACAACT<br>GAAATTCAAATCAACATTGAGATAGAATCTAGAAAATCCTACAAAAA<br>AATGCTAAAAGAAATGGGAGAGGTAGCTCCAGAATACAGGCATGAC<br>TCTCCTGATTGTGGGATGATAATATTATGTATAGCAGCATTAGTAATAA<br>CTAAATTAGCAGCAGGGGACAGATCTGGTCTTACAGCCGTGATTAGG<br>AGAGCTAATAATGTCCTAAAAAATGAAATGAAACGTTACAAAGGCTT<br>ACTACCCAAGGACATAGCCAACAGCTTCTATGAAGTGTTTGAAAAAC<br>ATCCCCACTTTATAGATGTTTTTGTTCATTTTGGTATAGCACAATCTTC<br>TACCAGAGGTGGCAGTAGAGTTGAAGGGATTTTTGCAGGATTGTTTA<br>TGAATGCCTATGGTGCAGGGCAAGTGATGTTACGGTGGGGAGTCTTA<br>GCAAAATCAGTTAAAAATATTATGTTAGGACATGCTAGTGTGCAAGC<br>AGAAATGGAACAAGTTGTTGAGGTTTATGAATATGCCCAAAAATTGG<br>GTGGTGAAGCAGGATTCTACCATATATTGAACAACCCAAAAGCATCA<br>TTATTATCTTTGACTCAATTTCCTCACTTCTCCAGTGTAGTATTAGGCA<br>ATGCTGCTGGCCTAGGCATAATGGGAGAGTACAGAGGTACACCGAG<br>GAATCAAGATCTATATGATGCAGCAAAGGCATATGCTGAACAACTCA<br>AAGAAAATGGTGTGATTAACTACAGTGTACTAGACTTGACAGCAGA<br>AGAACTAGAGGCTATCAAACATCAGCTTAATCCAAAAGATAATGATG<br>TAGAGCTTTGAGTTAATAAAAAA

Table S3. Comparison of our PEC biosensor with other reported works for DNA detection.

Analytical method	Detection limit	Linear range	Ref.
Fluorescence	3.5 pM	10 pM - 200 pM	[1]
Electrochemistry	72 aM	0.1 fM - 0.1 nM	[2]
Electrochemistry	5.2 fM	100 fM - 10 nM	[3]
Electrochemiluminescence	0.3 fM	3 fM - 0.3 nM	[4]
Photoelectrochemistry	9.0 fM	0.025 pM - 100 pM	[5]
Photoelectrochemistry	0.97 fM	1 fM - 10 pM	[6]
Photoelectrochemistry	37 aM	0.1 fM - 1 nM	[7]
Field-effect transistor	25 aM	1 aM - 10 fM	[8]
Surface-enhanced Raman scattering	3.1 fM	10 fM - 1 nM	[9]

References

[1] D. Fan, X. Zhu, Q. Zhai, E. Wang, S. Dong, *Anal. Chem.* **2016**, *88*, 9158.

[2] Q. Hu, Q. Wang, G. Sun, J. Kong, X. Zhang, *Anal. Chem.* **2017**, *89*, 9253.

[3] Y. Peng, Y. Huang, Y. Zhu, B. Chen, L. Wang, Z. Lai, Z. Zhang, M. Zhao, C. Tan, N. Yang, *J. Am. Chem. Soc.* **2017**, *139*, 8698.

[4] B. Babamiri, A. Salimi, R. Hallaj, *Biosens. Bioelectron.* **2018**, *117*, 332.

[5] C. Li, H. Wang, J. Shen, B. Tang, *Anal. Chem.* **2015**, *87*, 4283.

[6] X. M. Shi, L. P. Mei, Q. Wang, W. W. Zhao, J. J. Xu, H. Y. Chen, *Anal. Chem.* **2018**, *90*, 4277.

[7] H. Wang, M. Li, Y. Zheng, T. Hu, Y. Chai, R. Yuan, *Biosens. Bioelectron.* **2018**, *120*, 71.

[8] R. Campos, J. Borme, J. R. Guerreiro, G. Machado, J. M. F. Cerqueira, D. Y. Petrovykh, P. Alpuim, *ACS Sens.* **2019**, *4*, 286.

[9] Y. Liu, S. H. Wu, X. Y. Du, J. J. Sun, *Sensor. Actuat. B-Chem.* **2021**, *338*, 129854.
